# Reversible acetylation modulates dishevelled-2 puncta formation in canonical Wnt signaling activation

**DOI:** 10.1038/s41392-020-00229-0

**Published:** 2020-07-06

**Authors:** Jinhong Shen, Lin Hu, Li Yang, Mengshi Zhang, Weihong Sun, Xiaomei Lu, Gufa Lin, Chao Huang, Xiaoren Zhang, Y. Eugene Chin

**Affiliations:** 1 CAS Key Laboratory of Tissue Microenvironment and Tumor, Institute of Health Sciences, Shanghai Jiao Tong University School of Medicine & Shanghai Institutes for Biological Sciences, Chinese Academy of Sciences, University of Chinese Academy of Sciences, 200031 Shanghai, China; 2 Institutes of Biology and Medical Sciences, Soochow University Medical College, 215000 Suzhou, Jiangsu China; 3 Key Laboratory of Spine and Spinal Cord Injury Repair and Regeneration of Ministry of Education, Orthopaedic Department of Tongji Hospital, School of Life Sciences and Technology, Tongji University, 200065 Shanghai, China; 4 Clinical Medical Research Institute, First Affiliated Hospital of Xinjiang Medical University, 830054 Urumuqi, Xinjiang China

**Keywords:** Molecular biology, Cancer, Molecular biology, Cancer

**Dear Editor**,

Dishevelled-2 (DVL2) is a central adaptor protein that propagates signals from upstream receptors to downstream effector β-catenin in canonical Wnt signaling. At high local concentration, DVL2 assembles into signalosomes through DIX domain-mediated homo-polymerization to provide a docking platform for the recruitment of signaling components, which is critical for Wnt signaling transduction and activation;^1^ however, the exact underlying mechanism remains inconclusive. Although the roles of phosphorylation and ubiquitination in DVL2-puncta formation have been discussed before,^2^ the impact of lysine acetylation of DVL2 on this process remains elusive. Here we investigated the effect of acetylation on DVL2-puncta formation.

Exogenous DVL2-formed puncta that can aggregate into larger molecular signalosomes under treatment with Wnt3a or nicotinamide (NAM, SIRT deacetylases inhibitor) in Hela cells, suggesting the involvement of acetylation in DVL2 polymerization (Fig. 1a). Indeed, endogenous DVL2 was acetylated upon overexpression of Wnt3a in HeLa cells (Supplementary Fig. S1a). We further identified the acetyltransferase responsible for DVL2 acetylation. Among the acetyltransferases tested, only CREB-binding protein (CBP) that shuttles between cytoplasm and nucleus,^3^ exhibited acetylation induction activity toward DVL2 in cells (Supplementary Fig. S1b). Mass spectrometry was employed to identify K68 in DVL2 as a CBP-induced acetylation site, which was confirmed by loss of acetylation after site-directed mutation of lysine 68 to arginine (K68R) in DVL2 (Fig. 1b, c). Then a specific antibody targeting acetyl-K68 (aK68) of DVL2 (anti-DVL2 K68ac) was developed and verified (Supplementary Fig. S1c). Using this antibody, we confirmed Wnt3a-induced endogenous DVL2-K68 acetylation (DVL2-aK68) in HCT116 cells (Fig. 1d). However, DVL2-aK68 was reduced in CBP^−/−^ MEF cells, which cannot be enhanced by Wnt3a stimulation, further confirming the CBP-dependent DVL2 acetylation (Fig. 1e).

**Fig. 1:**
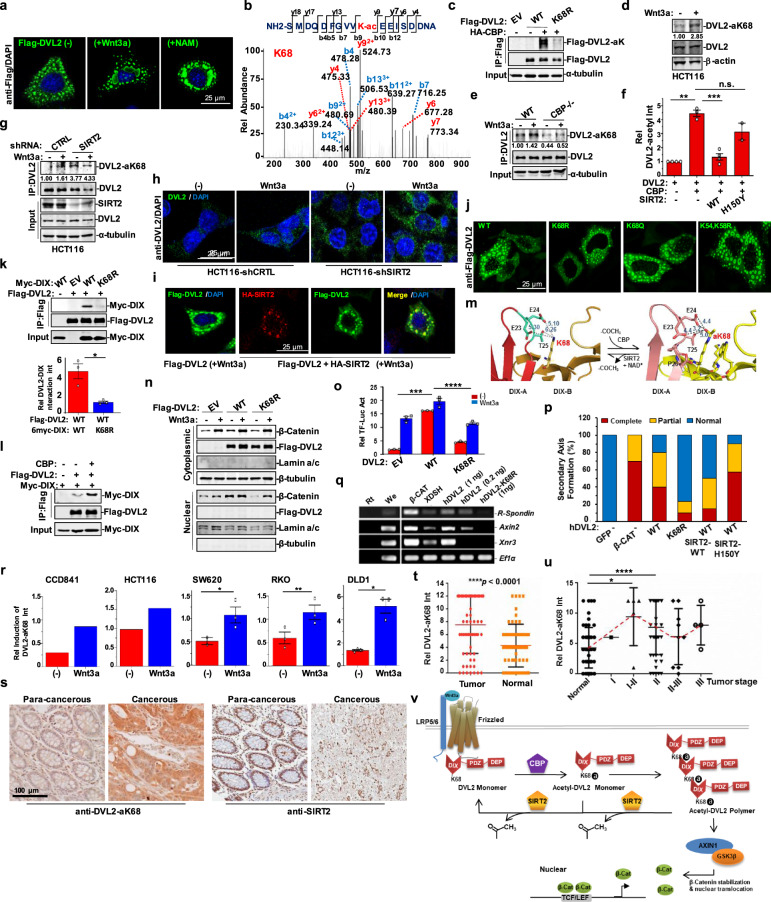
DVL2 acetylation at K68 promotes Wnt3a/β-catenin signaling activation through regulating DVL2-puncta formation and is associated with CRCs occurrence. **a** Immunofluorescence staining of Flag-DVL2 (green) in HeLa cells with or without indicated treatments for 2 h. The nuclei were stained using DAPI (blue). Data shown are representative of three independent experiments. **b** Mass spectrometric analysis of DVL2 acetylation on K68 residue. DVL2-immunoprecipitates from HEK293T cells transfected with DVL2 and CBP were digested into peptides with trypsin and subjected to mass spectrometric analysis. Data shown are representative of three independent experiments. **c** Immunoblot analyses for acetylation levels of Flag-tagged DVL2-WT and DVL2-K68R induced by CBP in HEK293T cells with pan acetyl-lysine antibody. EV represents for empty control expression plasmid. Data shown are representative of three independent experiments. **d** Immunoblot analyses for acetylation at DVL2-K68 (DVL2-aK68) in HCT116 cells with or without Wnt3a stimulation for 1 h using anti-DVL2 K68ac antibody. Data shown are representative of three independent experiments. The results were quantified by ImageJ software. The relative protein levels of DVL2-aK68 were normalized to β-actin levels. **e** Immunoblot analyses for aK68 levels of immunoprecipitated DVL2 from CBP^−/−^ and control MEF cells with or without Wnt3a treatment for 2 h using anti-DVL2 K68ac antibody. Data shown are representative of two independent experiments. The relative levels of DVL2-aK68 were normalized to immunoprecipitated DVL2 levels. **f** Deacetylation levels of immunoprecipitated DVL2 mediated by SIRT2-WT and SIRT2-H150Y mutant were analyzed by immunoblots with pan acetyl-lysine antibody. Densitometry of different independent experiments is shown. The results were quantified by ImageJ software. The relative levels of DVL2 acetylation were normalized to immunoprecipitated DVL2 levels. The results are shown as the means ± SEM. *p < 0.05, **p < 0.01, and ***p < 0.001 by two-tailed Student’s *t*-test. **g** Immunoblot analyses for aK68 levels of immunoprecipitated DVL2 from SIRT2-depleted and control HCT116 cells with or without Wnt3a treatment for 2 h using anti-DVL2 K68ac antibody. Data shown are representative of two independent experiments. The relative protein levels of DVL2-aK68 were normalized to immunoprecipitated DVL2 levels. **h** Immunofluorescence staining of DVL2 (green) in SIRT2-depleted HCT116 cells or control cells with or without Wnt3a treatment for 2 h. The nuclei were stained using DAPI (blue). Representative data are shown. **i** Immunofluorescence staining of Flag-DVL2 (green) in HeLa cells, with or without HA-SIRT2 (red) co-expression, under Wnt3a induction for 1 h. The nuclei were stained using DAPI (blue). Data shown are representative of two independent experiments. **j** Immunofluorescence staining of wild-type and variant mutants of Flag-DVL2 (green) in HeLa cells. Data shown are representative of three independent experiments. **k** Co-IP analyses for the interaction of full-length DVL2 and truncated-DIX variants expressed in HEK293T cells. The upper panel shows the representative immunoblot analysis, and the densitometry of three independent experiments is shown below. The co-immunoprecipitated DIX levels were normalized to immunoprecipitated DVL2 levels. Values are shown as the means ± SEM. *p < 0.05 by two-tailed Student’s *t*-test. **l** Co-IP analyses for the interaction of full-length DVL2 and truncated DIX domain with or without CBP co-expression in HEK293T cells. Data shown are representative of two independent experiments. The densitometry of two independent experiments is shown in Supplementary Fig. S3c. **m** Crystal structural model of the DIX–DIX interaction after artificially adding an acetyl group to K68 of DIX-B. DIX-A, means DIX domain A; DIX-B, means DIX domain B. The original structure file is 4WIP.pdb from database RCSB PDB (https://www.rcsb.org). **n** Cytoplasmic and nuclear levels of β-catenin in HEK293T cells transfected with wild-type or K68R-mutated DVL2 and control expression plasmids, with or without Wnt3a treatment for 2 h, were analyzed by immunoblot. Data shown are representative of three independent experiments. The densitometry of these independent experiments is shown in Supplementary Fig. S4a. **o** 293T cells were co-transfected with DVL2 variants or control expression plasmid, TOP/Flash reporter and Renilla luciferase normalization control, following with Wnt3a treatment for 12 h or not. Total cell lysates were collected to measure the firefly and Renilla luciferase activities. Values are the means ± SEM for each cohort (*n* = 3). *Adjusted *p*-value < 0.05, **adjusted *p-*value < 0.01, ***adjusted *p-*value < 0.001, and ****adjusted *p-*value < 0.0001 by two-way ANOVA. **p** Statistics of secondary axes formation in *Xenopus* embryos with mRNA microinjection of GFP (negative control and tracer), β-catenin (positive control), human DVL2 (hDVL2), hDVL2-K68R, or hDVL2 together with SIRT2-WT or SIRT2-H150Y mutant. 1 ng of mRNA of hDVL2 and variants was used in all injections. **q** RT-PCR detection of Wnt target genes in animal caps of *Xenopus* embryos after the indicated mRNA injection. EF1α served as loading control. **r** Immunoblot analyses for DVL2-aK68 levels in various colorectal cell lines treated with or without Wnt3a for 3 h. Data shown are densitometries of three independent experiments for SW620, RKO, and DLD1 cells, and two independent experiments for HCT116 and CCD841 cells. Values are shown as the means ± SEM. *p < 0.05 and **p < 0.01 by two-tailed Student’s *t*-test. **s** Representative images of DVL2-aK68 expression (left two) and SIRT2 levels (right two) based on immunohistochemistry analyses of tissue microarrays of human CRCs that comprising paired tumor and their adjacent normal tissues. **t** The general expression of DVL2-aK68 in CRC tissue microarrays was analyzed by two-tailed Student’s *t*-test, *n* = 67 pairs, *p < 0.05, **p < 0.01, ***p < 0.001, and ****p < 0.0001. **u** The expression of DVL2-aK68 in various CRC developmental stages was analyzed and compared by one-way ANOVA. *Adjusted *p*-value < 0.05, **adjusted *p*-value < 0.01, ***adjusted *p*-value < 0.001 and ****adjusted *p*-value < 0.0001. **v** Schematic model of DVL2 acetylation in regulation of canonical Wnt signaling. When Wnt3a binds to membrane receptors, DVL2 is recruited to Frizzled and then acetylated at K68 by nearby acetyltransferase CBP, forming DVL2 signalosomes. Following this, cytosolic β-catenin accumulates and translocates to the nucleus, triggering target genes expression. When signaling is significantly activated or terminated, deacetylase SIRT2 is recruited to DVL2 signalosomes and moves acetyl groups away from highly acetylated DVL2 gradually. Then DVL2 signalosomes are dissociated and Wnt signaling activity is downregulated.

To explore the role of CBP acetylation in DVL2-puncta formation, DVL2 and CBP were co-expressed (Supplementary Fig. S1d, right panel), or was expressed separately (Supplementary Fig. S1d, left panel), in HeLa cells. CBP colocalized with DVL2 and significantly promoted formation of DVL2 super-molecular puncta in cells (Supplementary Fig. S1d). Moreover, upon Wnt3a stimulation, endogenous CBP shuttled into cytoplasm from nucleoplasm to colocalize with endogenous DVL2 in HeLa cells (Supplementary Fig. S1e). Overall, these results indicate that CBP-dependent DVL2 acetylation has a role in DVL2-puncta formation.

Next, we investigated the enzymes responsible for DVL2 deacetylation. Treatment of cells with NAM, but not TSA (Trichostatin A, inhibitor of HDACs), significantly enhanced the levels of DVL2-aK68 regardless of Wnt3a stimulation (Supplementary Fig. S2a). These data suggest that one or more SIRT family members are responsible for DVL2 deacetylation. We thus tested effects of various SIRT proteins (SIRT1-7) on DVL2 deacetylation (Supplementary Fig. S2b). Among four SIRTs (SIRT1,2,4,5) that can deacetylate DVL2 when overexpressed in cells, SIRT4 and SIRT5 were excluded due to their mitochondrial localization. SIRT2, but not SIRT1, showed deacetylase activity toward DVL2-aK68 peptides that could be inhibited by NAM in vitro (Supplementary Fig. S2c). Consistently, SIRT2 catalytic mutant (SIRT2-H150Y) lost deacetylase activity on DVL2 (Fig. 1f). In addition, depletion of SIRT2 increased levels of DVL2-aK68 and DVL2 homo-oligomerization that can be further enhanced by Wnt3a stimulation to result in much bigger size of DVL2 aggregates in HCT116 cells (Fig. 1g, h). By contrast, both the size and number of Wnt3a-induced DVL2 macromolecular puncta were reduced upon co-expression of SIRT2 (Fig. 1i). These results indicate that SIRT2 is responsible for DVL2 deacetylation and may attenuate DVL2 homo-polymerization via deacetylation on K68.

Further, we analyzed the effect of DVL2-aK68 on puncta formation. Expression of DVL2-K68R mutant resulted in fewer puncta-containing cells that showed puncta with reduced size and number compared to cells expressing wild-type DVL2 (DVL2-WT) (Fig. 1j; Supplementary Fig. S3a). In contrast, other KR mutation did not significantly affect DVL2-puncta formation (Fig. 1j; Supplementary Fig. S3a). As K68 is right located on DIX domain of DVL2 and the DIX–DIX interaction is critical for DVL2 homo-polymerization^1^ (Supplementary Fig. S3b), we analyzed the impact of aK68 on DVL2–DIX homomeric interactions. Interaction between truncated Myc-tagged DIX domain and full-length Flag-DVL2 can be either interrupted by DVL2-K68R mutation (Fig. 1k), or enhanced by overexpression of CBP (Fig. 1l; Supplementary Fig. S3c). Crystal structural model of DIX–DIX interaction also showed that acetyl group added to K68 tightened their molecular bonding and stabilized the overall DIX–DIX conformation (Fig. 1m). Thus, aK68 in DIX domain may promote DVL2-puncta formation through directly strengthening DVL2 homomeric interactions conferred by DIX.

Upon Wnt activation, cytoplasmic β-catenin is stabilized and translocated to nucleoplasm to promote Wnt target genes expression. Compared to DVL2-WT, DVL2-K68R significantly decreased cytosolic accumulation and nuclear translocation of β-catenin (Fig. 1n; Supplementary Fig. S4a). Furthermore, TOP/Flash reporter assays showed that β-catenin transcriptional activity was strongly inhibited by DVL2-K68R, regardless of Wnt3a stimulation (Fig. 1o). Similarly, TOP/Flash activity induced by DVL2-WT, but not DVL2-K68R, was markedly enhanced by CBP with or without Wnt3a treatment (Supplementary Fig. S4b). These findings indicate that CBP-induced acetylation at DVL2-K68 is required for full activation of Wnt signaling by DVL2.

Ectopic activation of Wnt/β-catenin signaling in ventral vegetal blastomeres can induce formation of secondary dorsal body axes in early *Xenopus* embryos. As expected, injection of DVL2-WT mRNAs into ventral side successfully induced secondary axes formation in majority of the *Xenopus* embryos, similar to β-catenin injection that served as the positive control (Fig. 1p; Supplementary Fig. S4c, d). However, induction of secondary axis was significantly reduced by K68R mutation in DVL2 (Fig. 1p; Supplementary Fig. S4c, d). Interestingly, co-injection of SIRT2-WT, but not SIRT2-H150Y, partially reduced the numbers of embryos with DVL2-induced double axes (Fig. 1p; Supplementary Fig. S4d). In addition, the reduced expression of Wnt target genes (*Xnr3, Axin2*, and *R-spondin*) in the animal caps injected with DVL2-K68R also confirmed the inactivation of Wnt pathway by K68R mutation in DVL2 (Fig. 1q).

Constitutive activation of canonical Wnt signaling resulted from upregulated DVL2 is observed in colorectal cancers (CRCs).^4,5^ Considering the role of DVL2 acetylation in Wnt signaling activation, we explored the physiological significance of DVL2-aK68 in human CRCs. We detected low expression of DVL2-aK68 in CRC cell lines and one normal epithelia cell line CCD841, which could be strongly elevated by Wnt3a stimulation, especially in DLD1 cells (Fig. 1r; Supplementary Fig. S5a). Interestingly, upregulated CBP expression was also observed in the same cell lines (Supplementary Fig. S5a). Moreover, expression of DVL2-WT, but not DVL2-K68R, rescued reduced-rates of colony formation in DVL2-depleted FHC cells (Supplementary Fig. S5b). Immunohistochemistry analyses of tissue samples from human CRC microarrays showed that levels of DVL2-aK68 were significantly higher in CRC tumor tissues than that in the corresponding normal tissues (Fig. 1s, t). We further analyzed the relevance of DVL2-aK68 to the stages of CRC progression according to the clinic pathological characteristics of the patients. Levels of DVL2-aK68 were significantly increased during transition of normal to stages I–II and then maintained steady at later stages (Fig. 1u), indicating the association of DVL2-aK68 with the occurrence and initiation of CRCs. Moreover, SIRT2 expression in tumor was lower than that in adjacent para-cancerous tissues (Fig. 1s, right). Assessments of the clinical correlation between SIRT2 and CRCs based on the database Oncomine showed that SIRT2 expression was decreased in two randomly picked samples and stayed steady during tumor progression (Supplementary Fig. S5c). These analyses suggest that high levels of DVL2-aK68 in some CRCs may result from reduction of SIRT2, which acts as a tumor suppressor in multiple human cancers.

In conclusion, our work identified CBP-dependent DVL2 acetylation at K68 as a novel regulator in Wnt3a-induced DVL signalosome formation, which is critical for activation of downstream β-catenin signaling (Fig. 1v). Thus, DVL2-K68 acetylation may serve as a potential therapeutic target in clinical treatment of CRCs.

## Supplementary information

Supplementary Materials
