# Supplementary material for: Reversible acetylation modulates dishevelled-2 puncta formation in canonical Wnt signaling activation
Source: Signal Transduct Target Ther. 2020 Jul 6;5:115. doi: 10.1038/s41392-020-00229-0 (PMC7338394; doi:10.1038/s41392-020-00229-0)
Supplement: Supplementary file 1 — Supplementary Materials [file 41392_2020_229_MOESM1_ESM.docx]

Supplementary Materials for

**Reversible Acetylation Modulates Dishevelled-2 Puncta Formation in Canonical Wnt Signaling Activation**

Jinhong Shen^1, 2*^, Lin Hu^2*^, Li Yang^3^, Mengshi Zhang^3^, Weihong Sun^1^, Xiaomei Lu^4^, Gufa Lin^3^, Chao Huang^1#^, Xiaoren Zhang^1#^, and Y. Eugene Chin^2#^

^1^CAS Key Laboratory of Tissue Microenvironment and Tumor, Institute of Health Sciences, Shanghai Jiao Tong University School of Medicine & Shanghai Institutes for Biological Sciences, Chinese Academy of Sciences, University of Chinese Academy of Sciences, Shanghai 200031, China

^2^Institutes of Biology and Medical Sciences, Soochow University Medical College, Suzhou, Jiangsu 215000, China

^3^Key Laboratory of Spine and Spinal Cord Injury Repair and Regeneration of Ministry of Education, Orthopaedic Department of Tongji Hospital, School of Life Sciences and Technology, Tongji University, Shanghai 200065, China

^4^Clinical Medical Research Institute, First Affiliated Hospital of Xinjiang Medical University, Urumuqi, Xinjiang 830054, China

* These authors contributed equally.

Correspondence to:

Y. Eugene Chin^2^ (e-mail: [chinyue@suda.edu.cn](mailto:chinyue@suda.edu.cn);)

Xiaoren Zhang^1^ (e-mail: [xrzhang@sibs.ac.cn](mailto:xrzhang@sibs.ac.cn);)

Chao Huang^1^ (e-mail: [chaohuang@ynu.edu.cn](mailto:chaohuang@ynu.edu.cn);)

**This PDF file includes:**

Materials and Methods

Figures. S1 to S5

Materials and Methods

**Cell culture and transfection**

L-Wnt3a cell line was a kind gift from Dr. Xiaoren Zhang lab. HCT116-shCTRL and HCT116-shSIRT2 cells were kindly gifted from our colleague Dr. Yongxu Zhao. HCT116, RKO, DLD1, SW620 and CCD841 cells were kind giftes from Dr. Lixing Zhan lab.

L-Wnt3a cells were cultured using standard DMEM (HyClone, Logan, UT, USA) supplemented with 10% fetal bovine serum (FBS) (Gemini, CA, USA), 100 units/ml penicillin and 100 ug/ml streptomycin (Shenggong, Shanghai, China). MEFs, HEK293T cells were cultured in DMEM with 10% FBS, 100 units/ml penicillin and 100 ug/ml streptomycin. HeLa cells were cultured in RMPI-1640 (HyClone) containing 10% FBS, 100 units/ml penicillin and 100 ug/ml streptomycin. Colorectal cancer (CRC) cell lines HCT116, RKO, DLD1 and SW620 were cultured in DMEM with 10% FBS, while normal cell line CCD841 was cultured in RMPI-1640 containing 10% FBS. FHC cells (purchased from the ATCC) were maintained in RMPI-1640 supplemented with 10% FBS, 100 units/ml penicillin and 100 ug/ml streptomycin.

Plasmids were transfected using Lipofectamine 2000 as described by the manufacturer (Invitrogen, CA, USA).

**Plasmids, antibodies and reagents**

Wildtype Flag-DVL2, TOP-Flash luciferase reporter and Wnt3a-pCS3 plasmids were kind gifts from Dr. Lin Li of SIBS, CAS (Chinese Academy of Sciences). All mutants were generated using Site-Directed Mutagenesis method developed in our laboratory. Primers are shown in below.

DVL2-K68R-Flag:

F-5’ -TTCGGGGTGGTGAGGGAAGAAATTTCAGATGAC;

R-5’ -GTCATCTGAAATTTCTTCCCTCACCACCCCGAA;

DVL2-K68Q-Flag:

F-5’ -TTCGGGGTGGTGCAGGAAGAAATTTCAGATGAC;

R-5’ -GTCATCTGAAATTTCTTCCTGCACCACCCCGAA;

DVL2-K54,58R-Flag:

F-5’ -CGGCCCGCGGGCGCCAGGTACTTTTTCAGGTCTATGGATCAGGAT;

R-5’ -ATCCTGATCCATAGACCTGAAAAAGTACCTGGCGCCCGCGGGCCG.

All DIX truncated plasmids were subcloned from Flag-DVL2 and inserted into 6*MYC-tagged pcDNA3.0 vector. Primers are shown in below.

DIX-6*MYC:

F-5’- TCGCGGCCGCATGGTTGGGGAGACGAAGGTGATTTAC;

R-5’ -GATCTAGACTAATCTGAGGACACCAGCCAGGATACCAC.

The lentivirus plasmids for overexpressing DVL2 or its mutants were constructed in pMSCV-GFP vector. DVL2 small interfering RNAs (siRNAs) used for shRNA-DVL2 construction were generated from GenePharma Biotech (Shanghai, China). The lentivirus plasmids for DVL2 depletion were constructed in pLKO.1-puro vector. The shRNA sequences against DVL2 are noted below.

DVL2-shRNA #2597 oligo:

F-5’ -CCGGAACTGCCATACAGGAGATTGTCTCGAGACAATCTCCTGTATGGCAGTTTTTTTG- 3’;

R-5’ -AATTCAAAAAAACTGCCATACAGGAGATTGTCTCGAGACAATCTCCTGTATGGCAGTT- 3’.

Commercial antibodies of MYC or HA-Mouse tag, DVL2, SIRT2, β-catenin and Lamin a/c were all purchased from Santa Cruz Biotech (CA, USA). Antibodies of Flag-M2, actin and tubulin were obtained from Sigma-Aldrich (St. Louis, Missouri, USA). Antibodies of acetyl-lysine (pan), CBP and HA-Rabbit were purchased from Cell Signaling Technology (Danvers, Ma, USA). Specific polyclonal antibody recognizing acetyl-lysine 68 of DVL2 (anti-DVL2 K68ac) was prepared by Dr. Chuangui Wang and Dr. Shengping Zhang lab of Shanghai General Hospital, SHSMU. IRDye® 800CW and IRDye® 680LT secondary antibodies for western blotting were purchased from LI-COR, Inc (Lincoln, USA). Alexa Fluor® 488 and Alex Fluor® 568 secondary antibodies for immunofluorescence were obtained from Invitrogen (CA, USA). Anti-FLAG and anti-HA beads were from Sigma-Aldrich. T4 DNA ligase system was from Thermo Fisher Scientific (CA, USA). All restriction enzymes were from Thermo Fisher Scientific or New England Biolabs (Ipswich, MA, UK).

Recombinant Human Wnt3a Protein (R&D Systems) (Minneapolis, MN, USA) was reconstituted at 200 μg/mL in sterile PBS containing at least 0.1% human or bovine serum albumin (BSA) (Ameresco, USA). Inhibitors trichostatin A (TSA) and nicotinamide (NAM) from Sigma-Aldrich were suspended in DMSO (Sigma-Aldrich).

**Wnt3a** **conditioned medium preparation**

Wnt3a conditioned medium was prepared according to ATCC instructions for L-Wnt3a cells. The cells were split and diluted (1:10) in 10 ml culture medium in 10 cm Petri dishes, and grown for 4 days (approximately, till full confluence was reached). Following this, cell culture medium (first batch) was taken off using sterile filters. Fresh culture medium (10 ml) was added and cells were cultured for additional 3 days. Afterwards, the second batch was also collected using sterile filters. The cell-free first and second medium batches were mixed at a ratio of 1:1 as the Wnt3a conditioned medium and stored at 4°C or -20°C.

**Immunoblotting and immunoprecipitation (IP) analyses**

Cells were lysed in RIPA buffer (20-188, Millipore) containing inhibitors (1 mM PMSF, protease inhibitor cocktails, protease inhibitors and 5 mM NAM). Cell debris were removed by centrifugation at 4°C and 13,000 rpm for 15 min and a few of supernatant was kept as Input for SDS-PAGE. The rest of whole cell lysates (supernatant) were immunoprecipitated with the appropriate antibody and agarose beads overnight at 4°C. The beads were extensively washed with PBS buffer (with or without 0.1% NP40) before being boiled and then analyzed by western blotting.

The specific primary antibody recognizing DVL2-aK68 (anti-DVL2 K68ac) was prepared by our colleague Dr. Chuangui Wang and Dr. Shengping Zhang lab. The following primary antibodies were commercially obtained: anti-DVL2 (sc-8026, Santa Cruz Biotechnology), anti-SIRT2 (sc-28298, Santa Cruz Biotechnology), anti-CBP (#7389, Cell Signaling Technology), anti-β-catenin (sc-7963, Santa Cruz Biotechnology), anti-HA (sc-7392, Santa Cruz Biotechnology), anti-Myc (sc-40, Santa Cruz Biotechnology), anti-Flag (F1804, Sigma-Aldrich), anti-β-tubulin (T619, Sigma-Aldrich), anti-β-actin (A1978, Sigma-Aldrich), and anti-Lamin a/c (sc-7292, Santa Cruz Biotechnology).

Reagents used for immunoprecipitation were as followed: Recombinant Protein G-Sepharose™ 4 Fast Flow (17-0618-01, GE Healthcare), rProtein A Sepharose® Fast Flow (17-1279-03, GE Healthcare), Rabbit IgG (sc-2027, Santa Cruz Biotechnology), and Mouse IgG (sc-2025, Santa Cruz Biotechnology).

Cytosolic β-catenin levels were determined after depleting cadherin-associated β-catenin with Separation Buffer (10 mM KCl, 10 mM Tris, pH 7.5 and 2 mM EDTA) containing PMSF and protease inhibitors.

**TOP/Flash luciferase reporter assay**

HEK293T cells were cultured in 24-well dishes and transfected with 120 ng of DVL2 variants plasmids, 120 ng of TOP/Flash reporter plasmids and 10 ng of Renilla plasmids as an internal control using Lipofectamine 2000 (Invitrogen). 24 hr post transfection, cells were treated with Wnt3a conditioned medium for 12 hr and cell lysates were collected and prepared. Then the luciferase activities were detected with the Dual-Luciferase Reporter System (#07311, Promega, MN, USA). The value was used to measure the Wnt/β-catenin transcriptional activity.

**Mass spectrometric analysis of protein acetylation**

Immunoprecipitated Flag-tagged DVL2 from HEK293T transfected cells was separated using SDS-PAGE. Coomassie-blue-stained DVL2 bands were excised from the gel to digest with proteases (trypsin or chymotrypsin) (Thermo Fisher Scientific, CA, USA) and analyzed using mass spectrometry analysis with a Thermo LC-MS/MS system.

**Immunofluorescence microscopy**

The sterilized coverslips were inserted into the 24-well plates in advance, and cells were plated into the 24-well plate at the indicated cell density. After cells adhered, DVL2 or other mutants were transfected into cells for 24 hr. Following this, cells were treated with indicated stimulation for 1-2 hr or not and then washed with PBS and fixed with 4% paraformaldehyde. Cells were blocked with PBS containing 1% FBS and 0.3% Triton-X-100 for 1 hr. The primary antibodies used were as followed: anti-DVL2 (sc-8026, Santa Cruz Biotechnology), anti-CBP (#7389, Cell Signaling Technology), anti-HA (#3724, Cell Signaling Technology), anti-Flag (F1804, Sigma-Aldrich). The secondary antibodies were coupled to AlexaFluor®488 or AlexaFluor®680. Afterwards, the coverslips with cells were extracted and mounted on glass slides. The specimens were observed with Zeiss laser-scanning confocal microscope (LSM Meta 510).

**Tissue microarrays of colorectal tumors: immunohistochemistry (IHC) analysis and scoring**

Tissue microarrays of colorectal tumors (CGt No.: HCol-Ade150CS-01; Lot No.: XT15-009) were obtained from ShGnghGi Outdo Biotech CompGny (Shanghai, China). Immunohistochemistry (IHC) staining was done with the specific antibody anti-DVL2 K68ac. According to the general histochemical scoring method, the following parameters were used. The degree of immunostaining of indicated proteins was scored as 0, 1, 2, and 3 (from low to high). Staining area scores were defined as following: area <10%, score 0; 10-25%, score 1; 25~50%, score 2; 50~75%, score 3; 75~100%, score 4. The scores were multiplied for the two scoring parameters to get the total score, that is the signal intensity, for every single sample dot.

***In vitro* assay for SIRT deacetylation**

SIRT2 protein was purified from prokaryotes and quantitated by our lab colleague Dr. Yongxu Zhao. Reactions were incubated in assay buffer (500 mM Tris-HCl, pH 8.0, 1.3 M NaCl, 27 mM KCl, 10mM MgCl2, 10 mg/ml BSA). The reaction system was listed as follows: 1 μl peptide (1 μg/μl), 2 μl NAD (30 mM), 1 μl SIRT2 (1 μg/μl) and 14 μl H_2_O; incubated at 37°C for 30 min. Then the samples were desalted using Zip-Tip and detected with dot blotting.

***Xenopus* embryos and microinjection**

*Xenopus* laevis embryos were obtained using *in vitro* fertilization and staged according to the Nieuwkoop and Faber (NF) tables (Nieuwkoop and Faber, 1967). Embryos were deviled with 2% cysteine (Sigma, SL, USA), pH 7.8, and then cultured in 0.1×NAM. Expression constructs were subcloned from pDNA3.0 vector into pCS2 vector and linearized with Not I for *in vitro* mRNA synthesis with mMessenge mMachine (Ambion, Texas, USA). Two of the ventral vegetal blastomeres of 8-cell stage embryos were microinjected with 1 ng of hDVL2 (and variants) together with 0.25 ng of GFP mRNA as a tracer. Secondary axis induction was examined at neurula and tailbud stages. Three separate batches of *Xenopus* embryos were used.

**Animal cap assay and RT-PCR**

For RT-PCR detection of Wnt target genes in *Xenopus* animal caps, 4-cell stage embryos were injected with 1 ng of mRNA into the animal blastomeres and cultured to the stage 8.5. Animal caps were collected and incubated in 0.5x normal amphibian medium, till control embryos reached the stage 11.5, when endogenous Wnt activity peaks. Total RNAs were extracted from 5 animal caps with Trizol reagent (Gibco, NY, USA), treated with Turbo DNase I (Ambion, Texas, USA), and reverse-transcribed into cDNA with Superscript III reverse transcriptase kit (Invitrogen). PCR were performed with Taq DNA polymerase kit (Invitrogen), at the annealing temperature of 55˚C, 33 cycles. Primers used are listed below.

Xnr3:

F-5’ -CGAGTGCAAGAAGGTGGACA-3’;

R-5’ -ATCTTCATGGGGACACAGGA-3’;

R-Spondin:

F-5' -GATTACAGTCACTGCCAAGC-3';

R-5' -CCATACTGCCTCATACCTTC-3';

Axin2:

F-5' -TACTTTCTGCCTCCGCTTG-3';

R-5' -GGCTTGTAGTTTTTAGTCTCCTG-3';

EF1α:

F-5' -CCTGAACCACCCAGGCCAGATTGGTG-3';

R-5' -GAGGGTAGTCAGAGAAGCTCTCCACG-3'.

***Xenopus* photography and microscopy**

GFP was observed in live embryos using a Leica M165FC fluorescent dissecting microscope with an eGFP filter set. Images were captured using a Leica camera (Leica, Wetzlar, Germany) and processed using Photoshop software (Adobe, CA, USA).

**Statistical analysis**

Statistical analyses were performed using GraphPad Prism 6.0 (GraphPad Software, Inc.). Comparisons between two groups were performed by two-tailed Student’s t-test. For more than two groups, we used one-way ANOVA or two-way ANOVA with Tukey’s multiple comparisons test. The data were presented as means ± SEM/SD. For all statistical tests, *p*<0.05 was considered statistically significant. Statistical significance was indicated by asterisks (*).

Figure. S1.


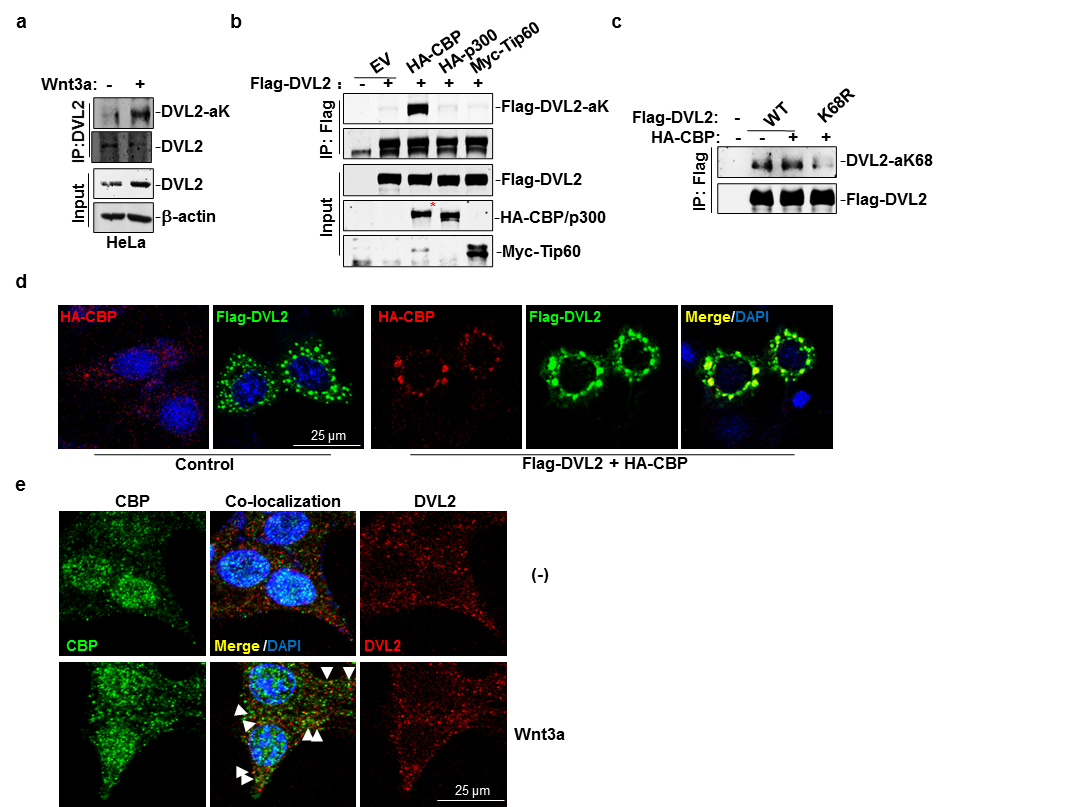


**Figure S1. DVL2 is acetylated at K68 by CBP upon Wnt3a stimulation.**

1. Immunoblot analyses of acetylation of immunoprecipitated DVL2 in HeLa cells transfected with Wnt3a plasmid or control expression plasmid.
2. Immunoblot analyses of acetylation of immunoprecipitated DVL2 in transfected HEK293T cells along with hemagglutinin (HA)-CBP, HA-p300 or Myc-Tip60. Red (*) was indicated for HA-CBP in Input.
3. Specific antibody test. DVL2 immunoprecipitants from HEK293T cells transfected with DVL2-WT or DVL2-K68R mutant were analyzed by immunoblot with an antibody specifically designed to recognize K68 acetylation site.
4. Immunofluorescence staining of Flag-DVL2 (green) in HeLa cells with or without HA-CBP (red) co-expression.
5. Immunofluorescence staining of endogenous DVL2 (red) and CBP (green) in HeLa cells with or without Wnt3a treatment for 2 hr.

Figure. S2.


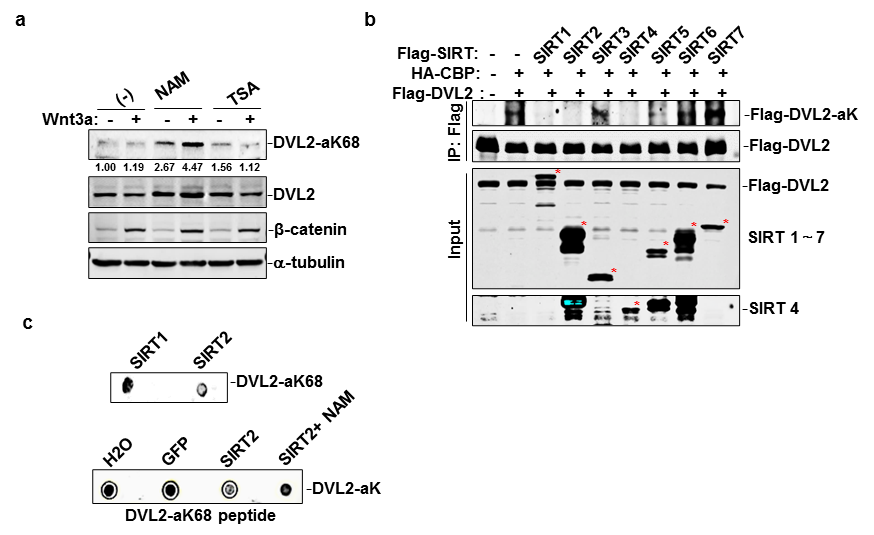


**Figure S2. DVL2 is deacetylated by SIRT2*.***

1. Immunoblot analyses of DVL2-aK68 in cytosol of HEK293T cells treated with NAM (10 mM) or TSA (1 mM) for 3 hr, and stimulated with or without Wnt3a for 1 hr in each group.
2. Immunoblot analyses for deacetylation of DVL2 in HEK293T cells transfected with SIRT family members.
3. *In vitro* assay for deacetylation of DVL2-aK68 peptide by SIRT1 or SIRT2 with dot blotting. H_2_O was used as the blank control for efficacy of the system and GFP was used as a negative control. NAM was used as the inhibitor of SIRT2 deacetylase.

Figure. S3.


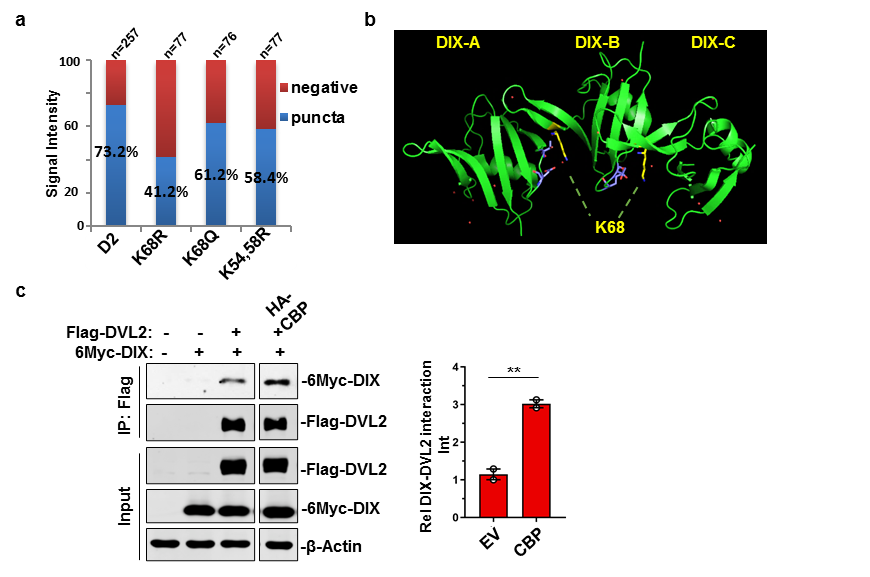


**Figure S3.**  **K68 acetylation promotes DVL2 homomeric interactions*.***

1. Statistics of relative ratio of cells containing DVL2 puncta against cells expressing DVL2 observed by immunofluorescence. Total cell counts (n) for each observation are shown. Data shown is representative for three independent experiments. The standard of DVL2 puncta was based on the cells containing the least (>=3) and smallest puncta among cells transfected with DVL2-WT.
2. Crystal structure model for three DIX-domains (A, B, C) of DVL2 crossed with each other in a head-to-tail mode. Original file 4WIP.pdb from RCSB PDB database was used for analysis.
3. The Co-IP analysis for the interaction between the full-length DVL2 and truncated-DIX with or without CBP co-expression in HEK293T cells (left panel). The densitometry of two independent experiments is shown (right panel). The levels of co-immunoprecipitated DIX levels were normalized to immunoprecipitated DVL2 levels. Values are shown as the means ± SEM. **p* < 0.05, ***p* < 0.01 by two-tailed Student’s t-test.

Figure. S4.


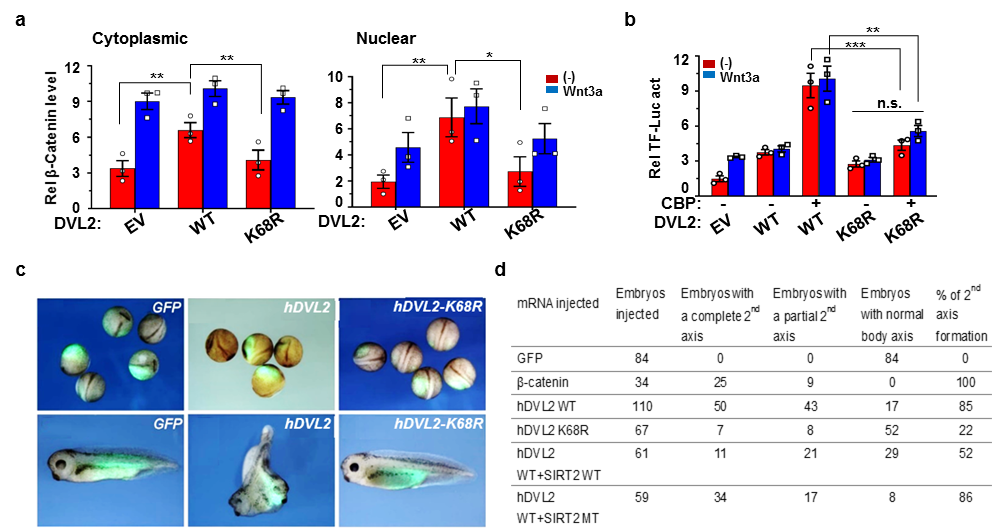


**Figure S4. Loss of DVL2 acetylation impairs Wnt3a signaling transduction and activation.**

1. The densitometry of the immunoblots for cytoplasmic and nuclear levels of β-catenin. The results were quantified by ImageJ software. The relative protein levels of β-catenin in the cytoplasm were normalized to β-tubulin levels, and in the nucleus were normalized to Lamin a/c levels. Values are the means ± SEM. *Adjusted *p* value < 0.05, **adjusted *p* value < 0.01 by two-way ANOVA. Representative experiment is shown in Fig. 1n.
2. CBP overexpression activated the TOP/Flash reporter luciferase activity in DVL2-WT but not DVL2-K68R transfected 293T cells. Cells were co-transfected with DVL2 variants, HA-CBP or control expression plasmid, together with TOP/Flash reporter and Renila luciferase normalization control following with Wnt3a treatment for 12 hr or not. 36 hr post transfection, total cell lysates were collected to measure the firefly luciferase and Renila activities. Values are means ± SEM for each cohort (n=3). *Adjusted *p* value < 0.05, **adjusted *p* value < 0.01 and *** adjusted *p* < 0.001 by two-way ANOVA.
3. K68R mutation in DVL2 diminished the induction of secondary axes in *Xenopus* embryos. Successful secondary axis induction by DVL2-WT was shown in pictures of the middle lane. Neurula stage embryos, upper row; tailbud stage embryos, lower row.
4. The summarized data of mRNA injection in ventral vegetal blastomeres in *Xenopus* embryos. mRNAs, indicated in the first column, together with 0.25 ng of GFP mRNA, were injected into the ventral vegetal blastomeres of 8-cell stage *Xenopus* embryos. Embryos were cultured until un-injected embryos reach tailbud stages. 1 ng of mRNA of hDVL2 and variants was used in all injections.

Figure. S5.


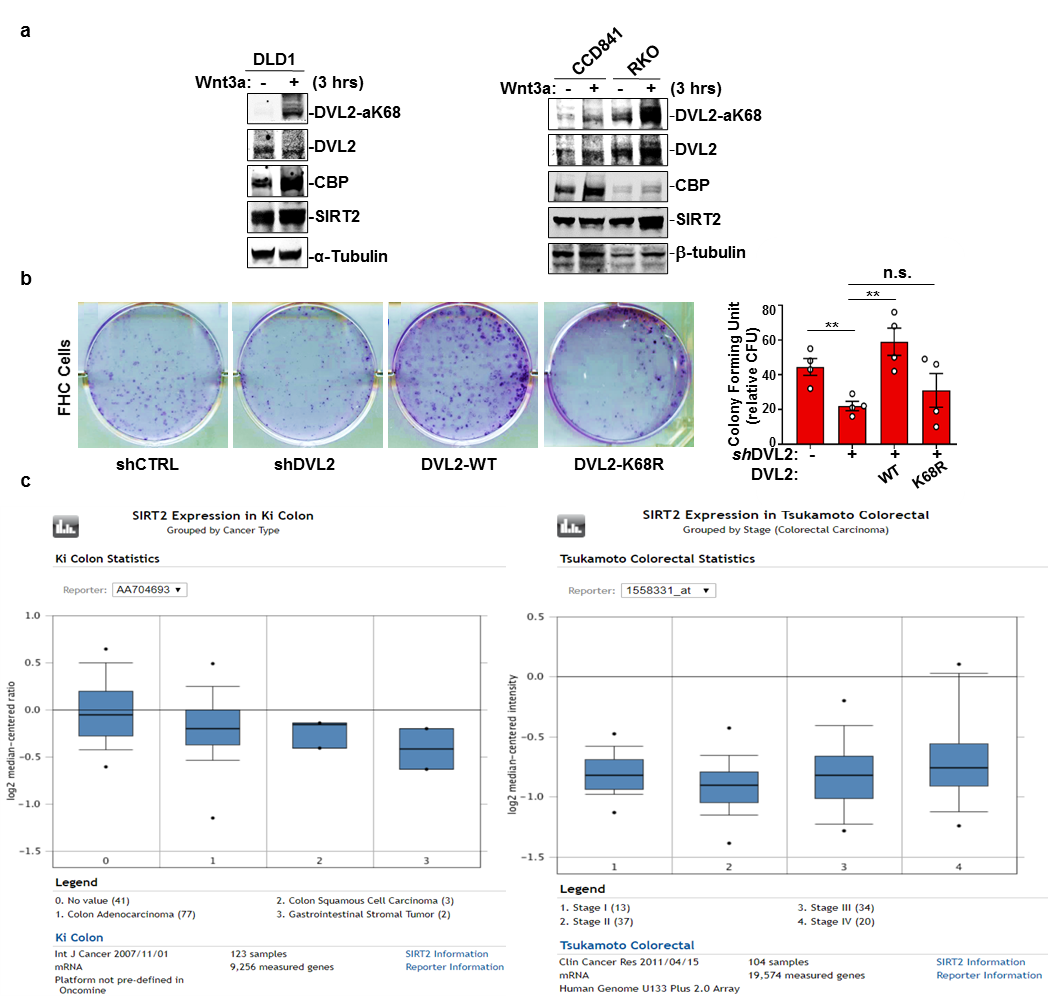


**Figure S5. DVL2 acetylation is positively relative to CRC progression*.***

1. Immunoblot analyses of DVL2-aK68 levels and corresponding levels of CBP and SIRT2 in DLD1, CCD841 and RKO cells.
2. Colony formation assays of control or DVL2-depleted FHC cells following with stable transfection of DVL2-WT or DVL2-K68R expression plasmid. Pictures shown are representative of three independent experiments. Statistical analysis was shown at right, **p* < 0.05 and ***p* < 0.01 by two-tailed Student’s t-test.
3. Analyses for clinical correlation of SIRT2 and human CRC in two samples obtained from open to public database Oncomine.
